# Lab-on-a-Chip Pathogen Sensors for Food Safety

**DOI:** 10.3390/s120810713

**Published:** 2012-08-06

**Authors:** Jeong-Yeol Yoon, Bumsang Kim

**Affiliations:** 1 Department of Agricultural and Biosystems Engineering, the University of Arizona, Tucson, AZ 85721, USA; 2 Department of Chemical Engineering, Hongik University, Seoul 121-791, Korea; E-Mail: bskim@hongik.ac.kr

**Keywords:** microfluidics, bioMEMS, food safety, water safety, *E. coli*, *Salmonella*

## Abstract

There have been a number of cases of foodborne illness among humans that are caused by pathogens such as *Escherichia coli* O157:H7, *Salmonella typhimurium*, *etc*. The current practices to detect such pathogenic agents are cell culturing, immunoassays, or polymerase chain reactions (PCRs). These methods are essentially laboratory-based methods that are not at all real-time and thus unavailable for early-monitoring of such pathogens. They are also very difficult to implement in the field. Lab-on-a-chip biosensors, however, have a strong potential to be used in the field since they can be miniaturized and automated; they are also potentially fast and very sensitive. These lab-on-a-chip biosensors can detect pathogens in farms, packaging/processing facilities, delivery/distribution systems, and at the consumer level. There are still several issues to be resolved before applying these lab-on-a-chip sensors to field applications, including the pre-treatment of a sample, proper storage of reagents, full integration into a battery-powered system, and demonstration of very high sensitivity, which are addressed in this review article. Several different types of lab-on-a-chip biosensors, including immunoassay- and PCR-based, have been developed and tested for detecting foodborne pathogens. Their assay performance, including detection limit and assay time, are also summarized. Finally, the use of optical fibers or optical waveguide is discussed as a means to improve the portability and sensitivity of lab-on-a-chip pathogen sensors.

## 1. Introduction

Food is one of the most important resources for humans, but unfortunately for microbes as well, resulting in many foodborne illnesses. The most common foodborne pathogens are *Escherichia coli* O157:H7, *Salmonella* spp., *Staphylococcus aureus*, *Campylobacter jejuni*, and *Listeria monocytogenes*, which have been found in many different food samples in the past couple of decades [1–4]. It has been estimated that *E. coli* O157:H7 and *Salmonella* pathogens alone have caused approximately cases of 1.4 million foodborne illness in the United States in 2010, with $2.7 billion in associated medical costs, productivity losses, and costs of premature deaths [5].

The lack of portable, real-time biosensors for these pathogens resulted in a significant time lag (a couple of days to a week) between the first outbreak and its identification. This time lag is caused by (1) delivering specimens to a remote laboratory (typically a day or two); and (2) subsequent analyses in the laboratory (taking a few hours up to a couple of days). In addition, the time and labor associated with these laboratory-based analyses have limited the number of sampling/analysis before outbreaks hit. In many cases, the remedy for pathogen contamination is not acted upon until there is an outbreak detected at the post-consumer level. If there were a means to speed up the tests by eliminating the need for an off-site laboratory, regular and routine testing would eliminate any pathogen caused problems before they reach the consumer level.

Current detection methods for foodborne pathogens include: (1) conventional culturing and colony counting; (2) enzyme-linked immunosorbent assay (ELISA); and (3) polymerase chain reaction (PCR) [6]. Culturing bacterial or viral pathogens is perhaps the oldest and yet the most accurate method. Certain bacterial pathogens can be cultured in a relatively simple manner, e.g., the sample liquid is diluted and spread on an agar plate, incubated for a day or two, and the number of colonies is counted. However, this is inappropriate for early detection since it takes days to see the results. ELISA can detect the pathogens much quicker, but its specificity is inferior to colony counting and PCR, because it requires multiple steps of reagent addition and rinsing. In ELISA, microwells are pre-immobilized with primary antibody, the sample liquid is added and rinsed, and secondary antibody, detector antibody-enzyme complex, and substrate are subsequently added and rinsed. The existence of target pathogen makes the enzyme-substrate complex not to be rinsed away, resulting in coloration. This is too complex to be used in field. PCR has similar complexity problems. In PCR, the target pathogen is lysed and its genetic material (DNA or RNA) is extracted. Primers, enzymes and other reagents are added to it, followed by cycling through three different temperatures and repeating the cycles 20–40 times. In this matter, the amount of a specific genetic sequence in a target pathogen can be amplified that can easily be detected by gel electrophoresis. It typically takes a couple of hours to finish the assay. These methods are not real-time detections in a strict sense. More importantly, all of these methods require skilled personnel to operate and a laboratory with expensive equipment.

Recently, numerous novel detection methods have been introduced that are capable of detecting pathogens in near-real-time, exhibiting excellent sensitivity and reproducibility, and have been made portable [7–11]. However, the criteria of what is ‘portable’ can vary considerably. A portable system may describe an apparatus that can be transported by one person, but is still rather bulky, weighing more than a few kilograms, and generally cumbersome to transport, especially in the field. The system may still require an AC outlet and/or a laptop computer for signal processing. Whereas, a handheld device should be defined as a single unit that is small enough to be held and operated by one hand, weighing as little as possible, preferably less than 1 kg. It should be battery-operated and have its own microprocessor, display, and integrated controls as well.

**Figure 1.**
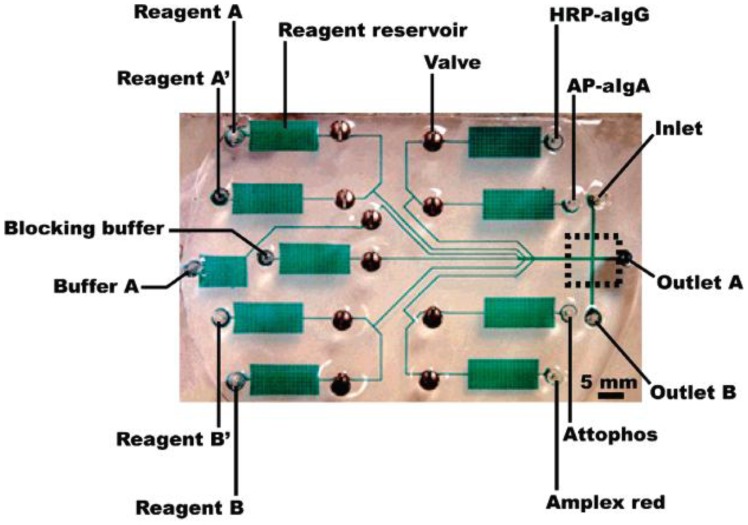
A lab-on-a-chip contains a network of channels and wells. Reprinted from [12] with permission © Am erican Chemical Society.

Lab-on-a-chip has recently been suggested as a perfect medium for portable and real-time medical diagnostics. Surely, there is no reason why we cannot use lab-on-a-chip for detecting foodborne pathogens. Lab-on-a-chip is a device that integrates several laboratory functions onto one small platform, typically only millimeters or centimeters in size. A lab-on-a-chip normally involves the handling of very small fluid volumes; this introduces the area of “microfluidics” that deals with the behavior, precise control, and manipulation of fluids that are constrained to a small, sub-millimeter scale. Lab-on-a-chip technology utilizes a network of channels and wells that are etched onto glass, silicon, or polymer chips to build mini-laboratories (Figure 1). Pressure or electrokinetic forces move small volumes in a finely controlled manner through the channels. Lab-on-a-chip enables sample handling, mixing, dilution, electrophoresis, staining, and detection on a single integrated system. The main advantages of lab-on-a-chip are: ease-of-use, speed of analysis, low sample and reagent consumption, and high reproducibility due to standardization and automation. In summary, the lab-on-a-chip-based biosensor is a perfect medium to make portable and real-time biosensing of foodborne pathogens possible.

In this review, we will summarize the requirements of lab-on-a-chip biosensor for field applications, highlight a group of lab-on-a-chip biosensors, and their applications to foodborne pathogens.

## 2. Requirements of Lab-on-a-Chip Biosensor for Field Use

**Figure 2.**
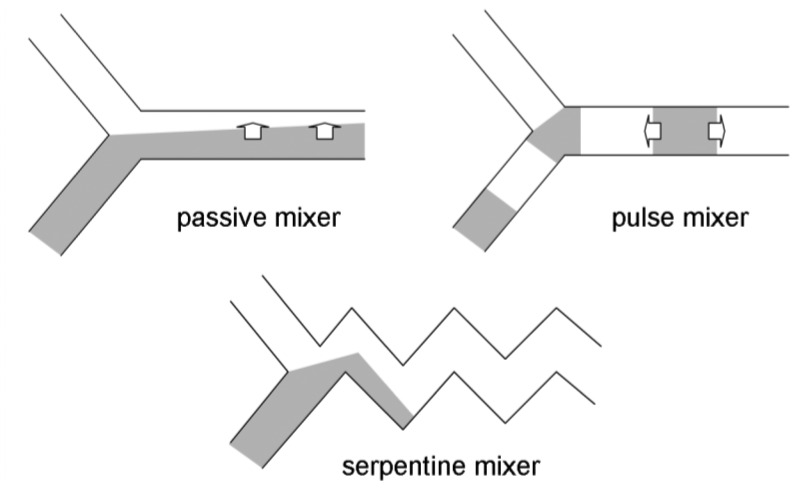
Microfluidic mixers [13]. Pure passive mixer: molecules diffuse to the other side purely by perpendicular diffusion. Pulse mixer: the fluid is supplied with pulse flow, allowing axial (*i.e.*, parallel to the flow) diffusion. Serpentine mixer: allows both perpendicular and axial diffusions.

**Figure 3.**
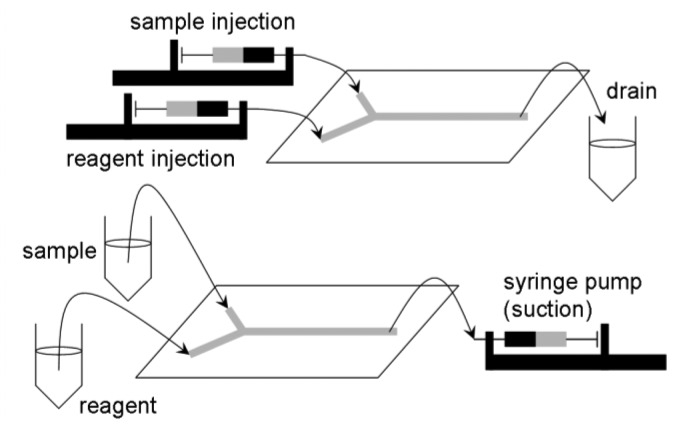
Sample/reagent injection and suction in immunoassay lab-on-a-chip devices.

**Figure 4.**
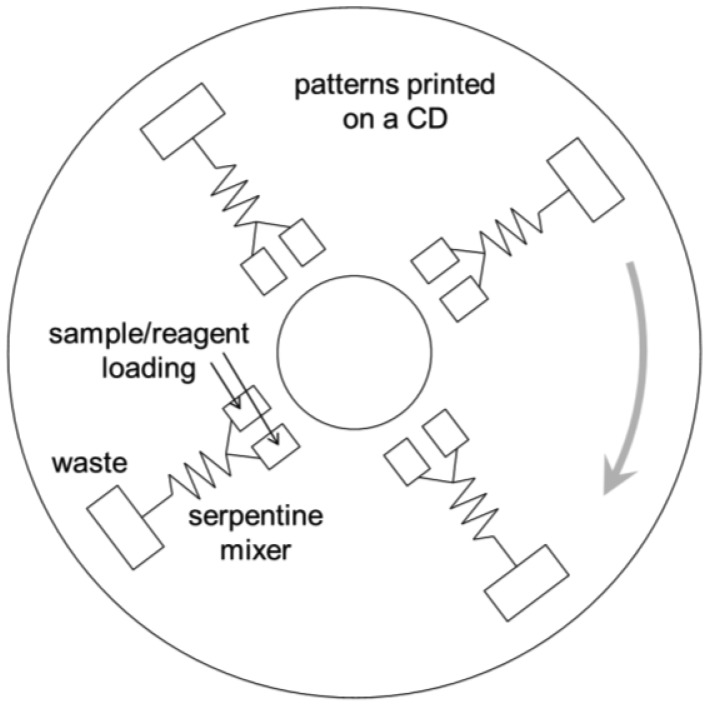
Lab-on-a-CD.

Lab-on-a-chip has primarily been investigated to replace the need for routine and high-throughput medical diagnostics. To use the lab-on-a-chip for field applications, the device should be made fully portable and capable of near-real-time detection. To this end, the following must be demonstrated:

1. *Automated liquid handling* (mixing, transport, and separation if necessary). This is one of the most-studied areas in lab-on-a-chip research. Y- or T-junction channels have been used to accomplish liquid mixing, coupled with several different designs of passive/pulse/serpentine mixer designs (Figure 2) [13]. In the past, more active mechanisms of microfluidic mixing have been suggested and tested, especially using microvalves and micropumps fabricated on chip [14]. Although these seemed promising and provided improved performance than the passive/pulse/serpentine microfluidic mixers, these are less common in pathogenic sensing due to the complications in fabrication and operation of devices that is inappropriate for complicated sample matrices such as food. Liquid transport is made by applying either voltage (electroosmotic flow) or external pressure (syringe pumping; Figure 3).
2. *Minimal sample pre-treatment*. When lab-on-a-chip is used in a diagnostic laboratory, sample pre-treatment is not as big of a problem since the necessary equipment is readily available in those environments. In field situations, however, pre-treatment could become very difficult. Food samples such as fruits and vegetables need to be ground and filtered/centrifuged to remove large debris (plant cells and tissue fragments). Food samples are sometimes simply washed with buffer since most pathogenic contaminations are found on the surface of food. For better performance, more rigorous sample pre-treatment steps become necessary: centrifuging multiple times, re-suspension of pellets with a vortex mixer and/or a sonicator, cell lysis/nucleic acid extraction (for PCR). These complicated procedures should be minimized into one or two simple steps using small and simple equipment such as a mini-centrifuge (battery-powered) or a syringe with filter, so that the sensor can be used by non-experts with minimal processing time.
  There have been numerous attempts to incorporate these sample pre-treatment processes into lab-on-a-chip. Centrifuging and membrane filtration are probably the most investigated, which are very important in dealing with food samples. One popular example of lab-on-a-chip centrifuging is lab-on-a-CD [15]. Microchannels are fabricated directly on the surface of a CD (compact disc), from its center to the outside, and the sample/reagent liquid is loaded to the inlet wells. This CD is then loaded into the CD player and rotated, creating a centrifugal force that makes the liquid to flow through the microchannel (Figure 4). Another example is the microfabrication of porous structure within microchannels, *i.e.*, on-chip fabrication of membrane filter [16].
3. *Fast*. A typical ELISA takes a few hours in a laboratory. A typical PCR (including cell lysis and gene extraction) also takes about a few hours in a laboratory. A normal cell culture and colony counting typically takes more than 24 hours. These times do not take the sample delivery time into consideration. “Real-time” detection, strictly speaking, indicates that the detection should be made simultaneously with sampling, but it would be safe to define less than 10-minute detection as real-time sensing. In some applications, however, real-time detection has been defined for the assays that can be finished in a single day, like 4–8 hours, and that are advertised under such conditions. We will not use this definition (4–8 hours as real-time) in this review, as 4–8 hours can hardly be accepted as real-time detections by general public.
4. *Fully integrated system*. The entire system should be incorporated into a single device, for the ease of use and equipment delivery. Many other biosensor systems require separate equipment for pre-treatment and/or detection. Almost all commercial biosensor systems (including lab-on-a-chips) require an external computer. A true fully-integrated system should not require any extra equipment. At minimum, it should have its own user interface (just a few buttons with no keyboard) and an integrated liquid crystal display (LCD) panel for system operation and displaying test results.
  If possible, the system should preferably have a data storage unit and/or data transmission system. The latter can be accomplished by using wireless protocols, such as Wi-Fi or cellular phone network (3 G or 4 G LTE). It is possible to use cellular phones for such data storage and transmission purposes, and in addition, smartphones can also be used for data processing or even as an optical detection system using its flash and camera.
5. *Battery-powered*. AC outlets are not always available in field situations. Therefore, the system should be operated fully with battery power. Low power consumption is necessary, which may prevent the use of electroosmotic flow (EOF; very common in lab-on-a-chip but requires relatively high voltage and power). Battery-powered lab-on-a-chips may not be very difficult to develop, although such demonstrations are relatively rare.
6. *No refrigerator required*. The reagents needed to complete the assays, such as the antibodies, nucleic acids, or enzymes, typically require refrigeration. In field situations, however, these reagents need to be packed in an ice box or lyophilized (freeze-dried) as powder for a possible storage in room temperature. This long-term storage study of reagents is relatively rare in biosensor and lab-on-a-chip studies.
7. Very low detection limit (i.e., *high sensitivity*). Detection limits (limits of detection) for common ELISA tests can be as low as tens of picogram proteins per mL of sample. Detection limits for common PCR can theoretically be at the level of single cell per 10–100 μL of sample, equivalent to 10–100 cells (normally represented by colony forming units; CFU) per mL of sample. About the same levels of detection limits are expected for lab-on-a-chips, but the actual limits have been a few or a few tens of nanogram proteins per mL of sample or a few hundred or million cells per mL of sample (10^2^–10^6^ CFU/mL) [17–21].

## 3. Immunoassay Lab-on-a-Chip with Optical Detection

ELISA is actually one type of immunoassays, where antibodies are used to capture the antigens in the target (pathogen). Therefore, it is possible to implement many different types of immunoassays, not just ELISA, in lab-on-a-chip platforms [22–27]. In this section, we will describe several different types of immunoassay lab-on-a-chips and their applications for foodborne pathogen detection.

### 3.1. Lateral Flow Assay (LFA)

There is a simpler format of immunoassays that gained a great popularity in diagnostics market, called lateral flow assay (LFA) or lateral flow immunochromatographic assay, or sometime simply dip-stick assay. The most well-known example of LFA is a pregnancy test. The user applies a drop of urine sample to a cassette-type device or dips the strip into a urine sample. If two pink lines show up, pregnant; one pink line, not pregnant; no line, test failure. LFA is essentially a membrane that is pre-loaded with a couple of different antibodies. This is considered as one of the simplest forms of lab-on-a-chip since liquid flows through the membrane by capillary action (thus microfluidic). LFA can certainly be used for detecting foodborne pathogens.

**Figure 5.**
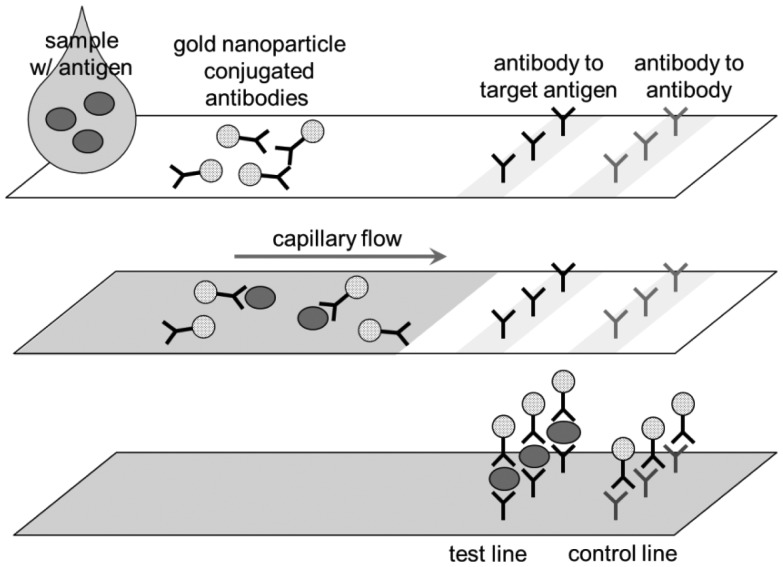
Schematic of LFA.

In LFA (Figure 5), there are two lines in the strip, one with antibodies to the target (e.g., anti-*E. coli*), the other with antibodies to antibody (anti-IgG). Once the sample solution (which may contain target, e.g., *E. coli*) is applied to the inlet, the anti-*E. coli*-gold nanoparticle (pre-loaded within the strip) may or may not bind to the target *E. coli*. The liquid travels through the membrane by capillary action. When the liquid hits the test line, the *E. coli* + anti-*E. coli*-gold nanoparticle complex is captured there. The unbound, excess anti-*E. coli*-gold nanoparticle continues to travel to the control line, which are eventually captured there. Gold nanoparticles absorb green light and some of blue light, so it looks pink. Therefore, two pink bands indicates the presence of target, one band the non-presence of target and no band the failure of assay (mostly because of not enough sample volume to achieve capillary action). Although gold nanoparticles (typically <40 nm [28]) are most frequently used in LFAs, colored or fluorescent latex particles (typically 100–900 nm [29]) are sometimes used.

The format shown in Figure 5 is called sandwich LFA, which is the most common form, but there are other types of LFAs. Competitive LFA is used when the target is of low molecular weight and has only one epitope (=antibody binding site), thus unable to form sandwich [30]. In competitive LFA, the sample is introduced together with the same targets that are labeled, and these unlabeled and labeled targets compete for the antibodies bound on the test line. This is less common in pathogen sensing since the targets are sufficiently big to form sandwich. Nucleic acid LFA utilizes a pair of primers (=short sequences of nucleic acids that are specific to the nucleic acids of the pathogens) instead of antibodies, and detect the presence of such nucleic acid sequences in the sample [30]. Since the amount of such nucleic acids in the sample can be very low, it typically requires PCR amplification (see Section 5). Due to this PCR requirement, nucleic acid LFA is less popular for field applications.

Nitrocellulose membrane is the most popular material used in LFAs. The pore size of such a membrane varies from 0.05 to 12 μm, which determines the speed of liquid transport and the sensitivity of the assay. The material choice and the pore size of membrane are particularly important when the sample has a high viscosity or contains fat globules (e.g., milk) [30,31].

LFA is very easy-to-use and inexpensive. It can be stored in room temperature. It is disposable, thus there is no cross-contamination issue. However, LFA is largely for yes/no assays and there is an issue of reproducibility. To address this weakness, the following techniques can be used: (1) spraying multiple test lines with varying antibody concentrations (semi-quantitative); (2) use of a reflectometer or a flatbed scanner, along with necessary software (typically comes with high price tag); or (3) readout by a cell phone camera (potentially very inexpensive but still under development). These quantitative LFAs provide sufficient sensitivity for many different clinical applications, but may not be sufficient for food safety applications. The reported detection limits of such systems are 10 ng/mL for IgG in mosquito bloodmeal [32], 0.2 ng/mL for *Schistosoma* in urine [33], 1 ng/mL for *Streptococcus pneumonia* in fish sperm [34], and 5 ng/mL for *Treponema pallidum* in serum [35].

### 3.2. ELISA Lab-on-a-Chip

**Figure 6.**
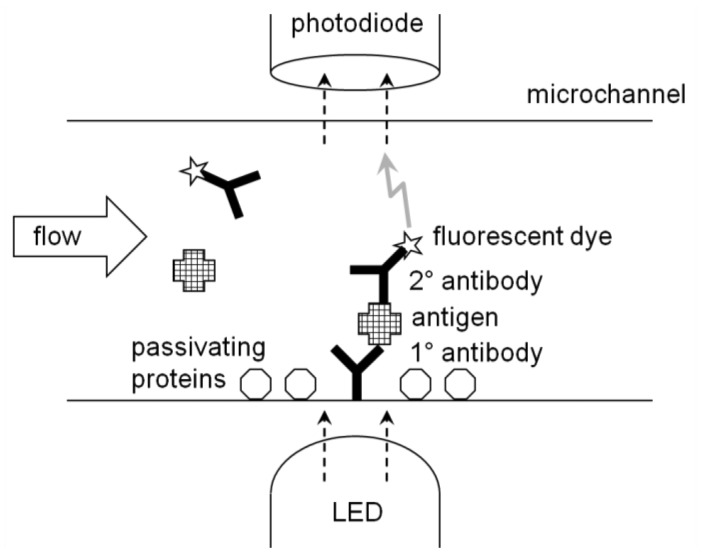
ELISA lab-on-a-chip.

ELISA is generally performed on a microplate (typically with 12 × 8 = 96 microwells in a single plate) and its result is quantified with a microplate reader. This is, in a way, a form of optical immunosensor. The wells can be replaced with a microchannel (thus lab-on-a-chip), where the target solution continuously flows through the microchannel (flow injection analysis). The antibodies are immobilized onto the inner surface, at a specific location, of a microchannel. The continuous flow's characteristics make the rinsing very effective. In fact, neither suction-dispensing cycle nor stirring is required, as the flow should remove any excess molecules from the inner surface of a microchannel. Light is irradiated to the location where the antibodies are immobilized; while the light sensor reads the signal from the other side of a microchannel (Figure 6).

Recently, numerous novel detection schemes have been demonstrated for foodborne pathogen detection using ELISA-type lab-on-a-chips, which are close to near-real-time with excellent sensitivity. Guan *et al.* [36] demonstrated the bioluminescence detection of *E. coli* O157:H7 (using anti-*E. coli*) with the detection limit of 3.2 × 10^1^ CFU/mL and the assay time of 20 min. Wojciechowski *et al.* [37] used the antibody-coated organic photodiode for the detection of Staphylococcal enterotoxin B with the detection limit of 0.5 ng/mL. Ricciardi *et al.* [38] used the antibody-coated microcantilever to detect *Salmonella* with the detection limit of 10^3^ CFU/mL and the assay time of 40 min. The use of antibody-conjugated magnetic particles for capturing pathogens in a microchannel has also been investigated. Schemberg *et al.* [39] used such magnetic particles in conjunction with fluorescent microcapsules for optically detecting single cells.

### 3.3. Surface Plasmon Resonance (SPR) Lab-on-a-Chip

Both LFA and ELISA lab-on-a-chip require secondary antibodies with “labels”, e.g., gold nanoparticles, fluorescent dye, and chemiluminescent dye, with subsequent multiple rinsing steps. There are several alternatives that do not involve “labels”, e.g., label-free immunoassay lab-on-a-chip. Demonstration of surface plasmon resonance (SPR) in a lab-on-a-chip platform is probably one of the most popular label-free immunoassay lab-on-a-chips.

**Figure 7.**
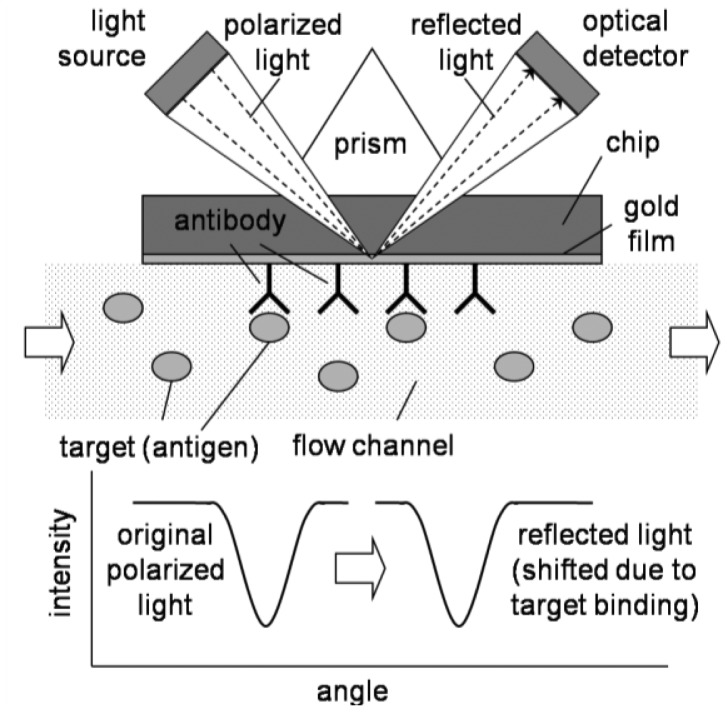
SPR lab-on-a-chip.

SPR is largely based on total internal reflection. As shown in Figure 7, light hits the metal-liquid interface, and it exhibits total internal reflection due to the difference of refractive indices of metal and liquid. In SPR, there are two important modifications: (1) the incident light is polarized; and (2) the surface is a thin metal film coating, usually gold [40,41]. Polarization means that the light is oscillating only at a certain orientation, where normal light oscillates at various orientations. When light hits the gold surface, electrons and holes are created (just like photodiode). Because the incoming photons are oscillating only at one orientation, the generation of electrons/holes (electric charges) will also be oscillated at one direction. This charge oscillation can propagate parallel to the gold film, called evanescent wave. Also this propagation is short-lived, thus it does affect the reflected light. For a certain angle of incident light, the incident light can be matched to this evanescent wave (resonated). If this resonance happens, the light intensity of reflected light at that angle will be greatly reduced.

The surface evanescent wave is a function of the refractive indices of gold and a liquid. If some molecules adsorb to the surface of gold film, the overall refractive index of the liquid near the gold film changes, as well as the resonance angle (Figure 7). In a typical SPR apparatus, this change of resonance angle (with reference to a bare surface) is shown as its sensor signal. This sensor signal is roughly equivalent to the mass of the adsorbed molecules. However, exact determination of the adsorbed mass is difficult in SPR sensors, as the refractive indices and other optical properties are quite different from molecule to molecule [40,41].

In an SPR immunosensor, the gold surface is pre-immobilized with antibodies, and the SPR signal with these antibodies will serve as a base line. The sensor chip is exposed to a flow channel to facilitate the introduction of target/reagent solutions and subsequent rinsing. The first commercial SPR-based biosensor was marketed by Biocore AB Corporation based in Sweden; and commonly used in many applications. Recently there have been many attempts to further miniaturize the entire system, especially miniaturizing the flow channel into a microchannel, thus lab-on-a-chip platform.

Fatima *et al.* [42] demonstrated the detection of antibiotics from milk using an SPR lab-on-a-chip, with the detection limit of 1.1–2.1 ng/mL in 5-fold diluted milk. Typical detection limits of SPR lab-on-a-chip for *E. coli* O157:H7 are 10^2^–10^3^ CFU/mL in milk, juice or beef [43], 10^4^–10^6^ CFU/mL [44], and 10^4^ CFU/mL in apple juice [45]. Typical detection limits of SPR lab-on-a-chip for *Salmonella typhimurium* are 10^4^ CFU/mL in apple juice [45] and 10^2^ CFU/mL [46]. In general, the detection limits of SPR lab-on-a-chips have been inferior to those of ELISA lab-on-a-chips.

### 3.4. Latex Immunoagglutination Assay (LIA) Lab-on-a-Chip

**Figure 8.**
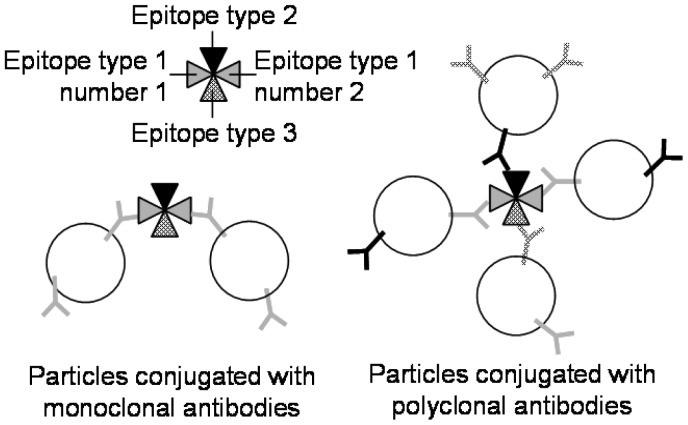
Latex immunoagglutination assay—formation of a doublet or bigger clumps through multiple epitope binding. Reprinted from [47] with permission © American Society of Agricultural and Biological Engineers.

There is another simpler format of immunoassay that can be reproducibly demonstrated in lab-on-a-chip, called latex immunoagglutination assay (LIA). Although this is not label-free, it is potentially rinse-free (both ELISA and SPR lab-on-a-chips require rinsing steps). In LIA, particles are coated with antibodies to a given target and mixed with the sample fluid in question. If the antigen is present in the fluid, the particles form larger aggregates (more precisely, agglutinates) due to antibody-antigen interactions, termed immunoagglutination (Figure 8) [47]. In the past, this has been detected visually, with the detection limit being the point at which agglutinates precipitate and become visible; however, measuring the immunoagglutination via forward light scattering in a lab-on-a-chip is more appropriate [48], as agglutinated submicron or nanoparticles are not required to precipitate out of solution for a positive signal [49,50]. Yoon's research group has primarily investigated LIA in lab-on-a-chip [51–56]. Particles with a diameter near the wavelength of visible light were used, *i.e.*, in the regime of Mie scattering, whose scattering intensity is not only very strong but also depends largely on the size and morphology of particles [57–59]. In the presence of target antigens, the microparticles agglutinate, effectively increasing the particle diameter, thus increasing the intensity of scattered light. In this way, a blank signal (antibody conjugated particles in solution without target) can be recorded and compared to scattering intensities from solutions with known concentrations of target antigen, and a standard curve can be constructed. Heinze *et al.* [58] detected avian influenza virus with the detection limit of 10 pg/mL in 1% diluted chicken feces. You *et al.* [59] detected *E. coli* with the detection limit of 10 CFU/mL in 10% diluted lettuce. Fronczek *et al.* [60] detected *Salmonella* with the detection limit of 10 CFU/mL in 10% diluted chicken tissue, all with the assay time of 10 minutes or less.

However, LIAs have not always provided perfect results. Specificity has always been a concern, as non-specific binding can lead to false-positive readings. To suppress non-specific binding, surfactants have been added to the particle suspension; however, the surfactants sometimes prevent the particles from agglutinating even when the target antigens are present (false-negative). To address this problem, highly carboxylated latex particles (*i.e.*, the surface was saturated with carboxyl side chains) may be used for LIA lab-on-a-chip since the highly carboxylated latex particles are able to enhance particle stability without the use of surfactants [49]. In addition, the use of highly carboxylated particles had an advantage in microfluidic mixing. In LIA lab-on-a-chip, the streams of particle suspension and target solution are mixed together in a Y-shaped microchannel as seen in Figure 2 or 3 to achieve a uniform mixture. Normally in a microfluidic condition, such mixing is very difficult because the flow is strictly laminar. However, highly carboxylated particles make this mixing possible in a microfluidic device via enhanced diffusional mixing [50]. Thus, micromixers within a microchannel (which can be difficult to fabricate and/or operate) are not necessary. Lucas *et al.* used highly carboxylated latex particles and showed neither false-positives nor false-negatives [51,52]. In addition, as it is impractical to ask users to conjugate antibodies, antibody-conjugated particles should be provided to end users. Since antibodies denature very easily in room temperature, especially when they are immobilized on a solid surface, they need to be refrigerated. Kwon *et al.* [56] demonstrated that antibody-conjugated, highly carboxylated particles can be stored in a refrigerator for up to 4 weeks without losing their activity. Antibodies were conjugated by typical covalent coupling (using carbodiimide) without use of surfactant. Furthermore, antibody-conjugated particles can even be lyophilized to a powder form, which can be stored in room temperature for several weeks, losing only 10% of scattering signals in respective assays. Fronczek *et al.* [60] demonstrated the lyophilization of antibody-conjugated particles within a microfluidic channel and subsequent LIA lab-on-a-chip for *Salmonella* with the detection limit of 10 CFU/mL.

## 4. Immunoassay Lab-on-a-Chip with Electrochemical Detection

### 4.1. Impedance Immunoassay Lab-on-a-Chip

**Figure 9.**
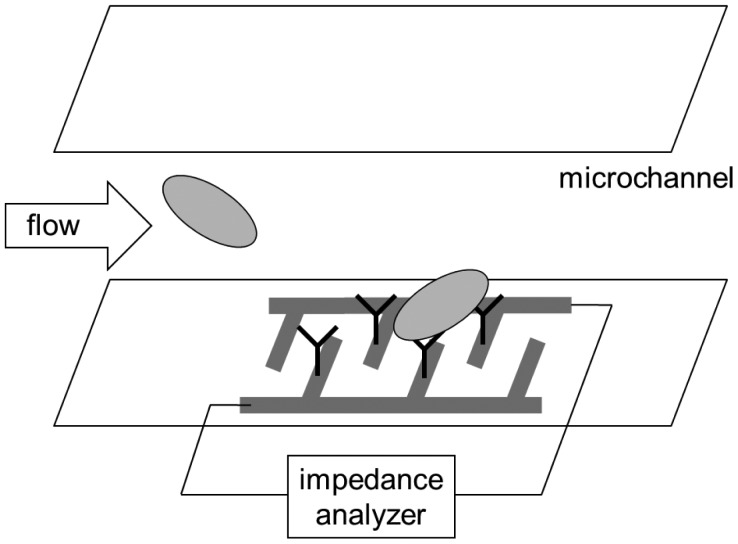
IME (interdigitated microelectrode) immunoassay lab-on-a-chip.

There is another type of “label-free” immunoassay lab-on-a-chip that utilizes electrochemical rather than optical detection. Impedance immunoassay lab-on-a-chip is probably the most popular example of such lab-on-a-chips (Figure 9). Antibodies are immobilized on a surface where two electrodes are patterned in a configuration called interdigitated microelectrode (IME). When a big target like bacterium binds to the surface-bound antibodies, some of the target may be able to bridge the two electrodes, thus lowering the resistance. If electrode patterns are made very small (microelectrode), then smaller targets such as viruses and proteins may be detectable.

To maintain the antibody-antigen binding and to prevent any possible oxidation/reduction, it is beneficial to apply alternating current (AC) rather than direct current (DC). An expanded version of resistance should be used with AC power, called impedance. Impedance is described as a complex number: *Z* (impedance) = *R* (resistance) + *jX* (reactance).

IME immunosensors have been successfully demonstrated for detecting pathogens in many different food sample matrices, including *E. coli* with typical detection limit of 10^4^–10^6^ CFU/mL from lettuce [61] and *Salmonella* with the single-cell detection limit from milk with 2–9 hours (*i.e.*, with culturing) of assay time [62]. Guo *et al.* [63] used the electrochemical impedance spectroscopy to detect *E. coli* with the detection limit of 10^2^ CFU/mL. Again, these detection limits are still inferior (or the assay time is very long) to those of “labeled” ELISA lab-on-a-chips.

### 4.2. Carbon Nanotube (CNT) Immunoassay Lab-on-a-Chip

The detection limits of “label-free” immunoassay lab-on-a-chips (SPR- and impedance-based) for food samples are typically inferior to those of “labeled” ELISA lab-on-a-chips. There have been several attempts to improve the sensitivity of electrochemical immunoassays in a lab-on-a-chip platform, and it looks like carbon nanotube (CNT)-based immunoassay lab-on-a-chip shows the most promising result. The main advantages of CNTs in electrochemical immunoassay are enhanced electronic properties, a large edge plane/basal plane ratio, and rapid electrode kinetics. Therefore, CNT-based immunoassays generally have higher sensitivities, lower detection limits, and faster electron transfer kinetics than traditional carbon electrodes.

Two types of CNTs are available, single-walled (SW) and multi-walled (MW). MWCNTs usually have 2–100 nm diameter (typically 2–10 nm in internal diameter), while SWCNTs usually have about 0.2–2 nm in diameter. CNTs have a high surface area to weight ratio of 300 m^2^/g, and most of this surface area is accessible to both electrochemistry and immobilization of biomolecules. The superior mechanical and conductive properties (high actuating stresses, low driving voltages, and high energy densities) of CNTs make them very promising for developing ultrasensitive and miniaturized immunosensors for disease diagnosis. It is expected that ELISA can be miniaturized and fabricated in the form of CNT-array-based sensor chips through NEMS (nano-electro mechanical system) techniques. However, CNTs themselves do not provide any measurable signals for sensing biomolecules. It is, therefore, of critical importance to develop techniques that can endow the CNTs with both a molecular recognition and a signal transduction function.

Garcia-Aljaro *et al.* [64] reported carbon nanotube (CNT)-based immunosensors for *E. coli* O157:H7 (bacterium) and T7 bacteriophage (virus). The transduction element consisted of SWCNTs, which were functionalized with specific antibodies, aligned in parallel bridging two gold electrodes to function as a chemiresistive biosensor. The detection limits are 10^3^–10^5^ CFU/mL for *E. coli* O157:H7 and 10^3^ PFU/mL for T7 bacteriophage. Yang *et al.* [65] combined CNTs, enhanced chemiluminescence and a cooled charge-coupled device (CCD) detector to improve the detection of Staphylococcal enterotoxin B (SEB) in food. Anti-SEB primary antibodies were immobilized onto the CNT surface, and the antibody-nanotube mixture was immobilized onto a polycarbonate surface. SEB was then detected by an ELISA assay on the CNT-polycarbonate surface with an enhanced chemiluminescence assay. SEB in buffer, soy milk, apple juice, and meat baby food was assayed with a detection limit of 0.01 ng/mL using the CCD detector, which was more sensitive than the conventional ELISA. They also developed lab-on-a-chip using this CNT-ECL immunoassay to detect SEB [66,67]. Hu *et al.* [68] reported an electrochemical immunosensor built on 3-D CNT-conducting polymer network for detection of hepatitis B surface antigen in human serum, reaching a detection limit of 0.01 ng/mL with a dynamic range of 5 orders of magnitude.

Detection limits of the above lab-on-a-chip immunoassays are summarized in Table 1.

## 5. PCR Lab-on-a-Chip

**Figure 10.**
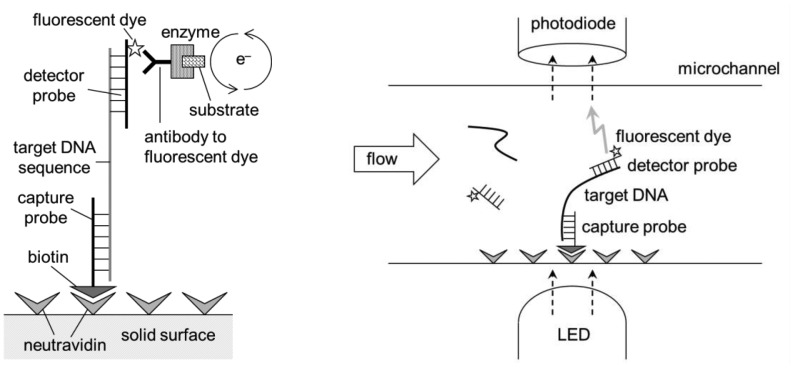
**Left**: A short sequence of DNA captures a target, complementary sequence, and a subsequent signal is detected in a manner similar to ELISA. **Right**: The same is demonstrated in a microfluidic channel in a manner similar to ELISA lab-on-a-chip.

Immunoassay-based lab-on-a-chips detect the presence of antigens on or from the pathogens. Binding of antibodies to their corresponding antigens are highly specific, but it is not always perfect. Cross-binding is common; for example, anti-*E. coli* often binds to other similar bacteria, such as *Salmonella*. A certain sequence of DNA or RNA can be used to detect its complementary sequence from pathogens, just like ELISA and ELISA lab-on-a-chip, where primary and secondary antibodies are replaced with capture and detector probes (short DNA or RNA sequences) (Figure 10). This sandwich DNA assay lab-on-a-chip provides much more specific assay results [69–73].

Due to their relatively low concentrations, however, it is necessary to amplify their amount using the process called polymerase chain reaction (PCR). As PCR is essentially a thermocycling process over three different temperatures (denaturing at 94–96 °C, annealing at 50–65 °C, and extending at 72 °C), it could be an attractive topic for lab-on-a-chip study. PCR labs-on-a-chip can be classified into three categories: (1) stationary chamber; (2) microchannel; and (3) droplet based, as discussed below [74].

### 5.1. Stationary Chamber PCR Lab-on-a-Chip

In a stationary chamber PCR lab-on-a-chip, nano- or even picoliter sized chamber(s) is patterned on lab-on-a-chip, and it is heated at three different temperatures to achieve thermocycling. This volume is substantially smaller than those of conventional PCR (typically on the order of microliters), which enables faster heat transfer thus leading to faster assays.

In addition, various sample preparation protocols can be incorporated in a lab-on-a-chip platform (together with PCR thermocycling), including: (1) pathogen capture, filtration and concentration, (2) cell lysis and DNA/RNA isolation, and (3) optical or electrochemical detection of amplified products (amplicons). For pathogen applications, Lee *et al.* [75] used magnetic beads for direct cell lysis and DNA capture, and Cheong *et al.* used optothermal properties of nanoparticles for pathogen lysis [76], both on integrated lab-on-a-chip PCR systems. On-chip detection is typically by fluorescence (*i.e.*, optical), but electrochemical detection is also possible [77,78]. It is sometimes necessary to separate amplicons and primers by on-chip electrophoresis to collect signals only from the amplicons [78].

### 5.2. Microchannel PCR

**Figure 11.**
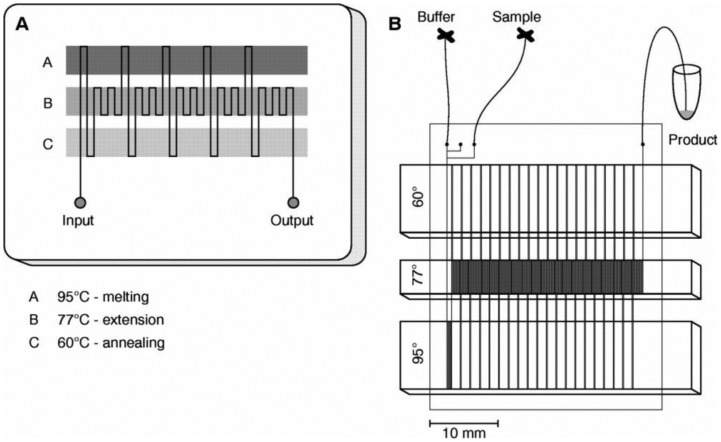
Continuous-flow PCR on a chip. Reprinted from [80] with permission © American Association for the Advancement of Science.

In conventional PCR, a single tube is repeatedly heated up and cooled down to achieve the desired temperatures. The environment for a tube, such as a Peltier plate, should be fully heated up and cooled down to make the tube to reach at desired temperature. As this process is primarily based on conduction heat transfer, which is the slowest among the three heat transfer mechanisms (conduction, convention and radiation), it often takes a couple of minutes to complete a single cycle (and thus over an hour to finish the typical 20–40 cycles of PCR). In a stationary chamber PCR lab-on-a-chip, the chamber is made very small to shorten this heating and cooling time. A better alternative is to move this liquid over three different temperature areas within lab-on-a-chip, so that the time required for heating and cooling may be significantly reduced, thus leading to a faster PCR assay. The first such demonstration was made by Kopp *et al.*, where a single serpentine microfluidic channel travels through three different temperature zones to achieve 20-cycle PCR. In this manner, <20-min or even <10-min 20–40-cycle PCR has become a possibility. Figure 11 shows the continuous-flow PCR on a chip made by Kopp *et al.* [79].

This concept has been combined with microfluidic cell lysis [80], capillary electrophoresis to confirm PCR products [78,81], or fluorescence microscopy for real-time monitoring of PCR products [82]. Liquid flows through a microchannel continuously, or as discrete liquid plugs within a microchannel. Li *et al.* [83] showed an integrated microfluidic RT-PCR method combined with online fluorescence detection by fluorescence microscopy for fast detection of rotavirus. Using this integrated device, they could amplify and detect rotavirus RNA within less than 1 hour with the RNA concentration of 3.6 × 10^4^ copies/μL. Delibato *et al.* [84] used a microfluidic-based capillary electrophoresis system to a PCR protocol for detecting *Listeria monocytogenes* in food and compared with a PCR with classical gel electrophoresis. The microfluidic electrophoresis PCR showed a relative accuracy of 100% with ISO (International Organization for Standardization) reference method, while the classical method showed down to 96%.

The problem of these attempts is that the user cannot change the assay protocol easily once the sample is introduced. In addition, the heat transfer throughout the microchannel device makes the heat isolation (required for thermocycling) very difficult, resulting in poor assay results.

### 5.2. Droplet PCR

The above problem can be resolved if PCR is demonstrated in a “digital” microfluidics format, where independent droplets move over a flat surface. Several methods have been suggested for digital microfluidics, electrowetting-on-dielectric (EWOD) [85], surface acoustic wave (SAW) [86], magnetofluidics [87], and wire-guide [88].

In EWOD, checkerboard-shaped electrodes are patterned on a surface with dielectric (also hydrophobic) coatings on top of it, and a droplet sits on one of such electrodes. If electricity is applied to one of the nearby electrodes, the surface becomes hydrophilic (thus electrowetting) due to the electric charge accumulated on it, and the droplet moves towards that electrode. In SAW, a droplet sits in between two interdigitated transducers (IDTs) that are patterned on piezoelectric material (e.g., quartz). Applying alternating current (AC) to IDT makes the material to compress and expand, creating surface acoustic wave. This SAW propagates the surface like an ocean wave, and able to make a droplet to move along the wave propagation. Creating IDTs arranged in X- and Y-directions enables two-dimensional movement of a droplet possible. In magnetofluidics, a droplet sits on top of a hydrophobic surface (typically superhydrophobic), with magnetic particles in it. The movement of a permanent magnet underneath the surface makes the magnetic particles to respond, eventually making the droplet to move along the permanent magnet's movement.

**Figure 12.**
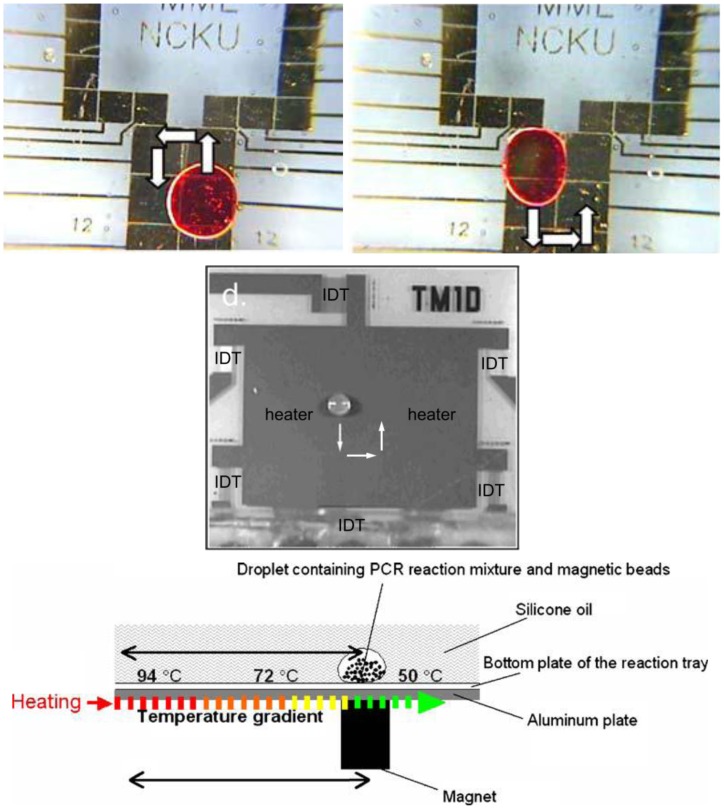
PCR demonstrations in digital microfluidics: EWOD (top), SAW (middle), and magnetofluidics (bottom). Reprinted from [89] and [91] with permission © Springer. Reprinted [90] with permission © Royal Society of Chemistry.

For all three cases, PCR thermocycling can be achieved by creating three different temperature zones on the device and the droplet cycles through these three zones multiple times (typically 20–40 times) to amplify the product, as seen in Figure 12.

Chang *et al.* [89] finished 25 EWOD-PCR cycles in 55 minutes for amplifying 511-bp target in a 15 μL droplet. Guttenberg *et al.* [90] completed 30-cycle SAW-PCR cycles in 10 minutes for amplifying 150-bp target in a 0.2 μL droplet. Ohashi *et al.* [91] finished 30 magnetofluidic-PCR cycles in 11 minutes for amplifying 126-bp target in a 3 μL droplet. Despite these achievements, EWOD-PCR and SAW-PCR have fabrication/operation complications and magnetofluidic-PCR has poor amplification issue arising from the presence of magnetic particles inside the droplet.

**Figure 13.**
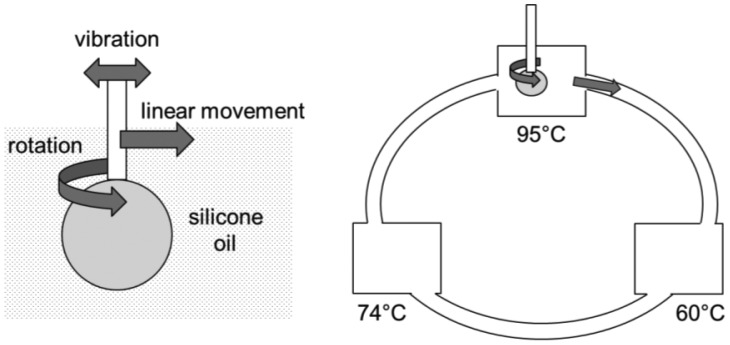
Wire-guided droplet PCR.

A better, yet simpler droplet-based PCR method has been recently proposed, called *wire-guided droplet PCR* [92] (Figure 13). Rather than using patterned electrode or a magnet/superhydrophobic surface combination, it uses a wire or a syringe needle to guide the movement of a droplet. The droplet moves over three silicone oil baths, where it is also vibrated and rotated to achieve better mixing and faster convective heat transfer. All other lab-on-a-chip PCR demonstrations use either conductive heat transfer or very limited convective heat transfer mechanisms. This method provides much faster thermocycling than the other droplet microfluidic PCR assays, typically less than 5–10 min, and allows easy incorporation of other procedures necessary for PCR assays, such as gene extraction, reverse transcription, and real-time quantification, *etc*.

### 5.3. Isothermal PCR in Lab-on-a-Chip

Typical PCR requires thermocycling over three different temperatures, which potentially makes fabrication and operation of PCR lab-on-a-chip quite complicated. As an alternative, various “isothermal” nucleic acid amplification methods (*i.e.*, only one temperature is needed) have been proposed, which is a good fit for field applications.

**Figure 14.**
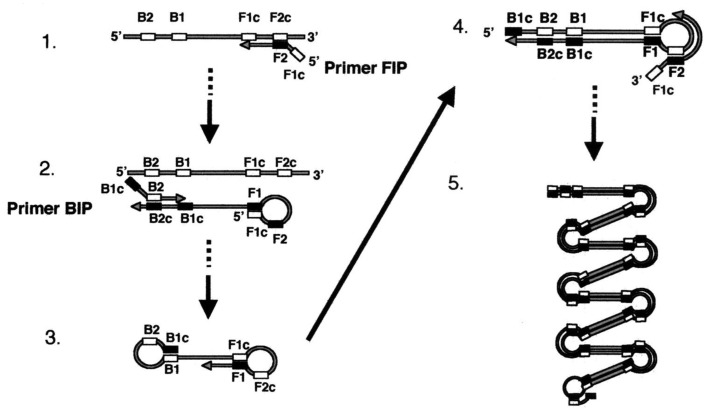
Loop-mediated isothermal amplification (LAMP). Reprinted from [94] with permission © American Society for Microbiology.

One example of such isothermal nucleic acid amplficiation methods is Loop-mediated AMPlification (LAMP; Figure 14) [93,94]. LAMP is popular since it requires only a single enzyme (strand displacing polymerase) and no preliminary manipulations to build a molecular motif capable of continuous self-replication. The LAMP technique is based on the principle of a strand displacement reaction and the stem-loop structure that amplifies the target gene fragment under isothermal conditions. LAMP is different from PCR in that four or six primers perform the amplification of the target gene, the amplification uses a single temperature step at 60–65 °C for about 60 min, and the amplification products have many types of structures in large amounts. Thus, LAMP is easier to perform than PCR, as well as being more specific. Furthermore, gel electrophoresis is not needed, because the LAMP products can be detected indirectly by the turbidity that arises from a large amount of by-product, pyrophosphate ion, yielding an insoluble white precipitate of magnesium pyrophosphate in the reaction mixture. Since the increase in the turbidity of the reaction mixture according to the production of precipitate correlates with the amount of DNA synthesized, real-time monitoring of the LAMP reaction can be achieved by real-time measurement of turbidity.

In recent years, there have been an increase number of papers, in the field of using isothermal nucleic acid amplifications (including LAMP) as a novel nucleic acids amplification technique for identifying pathogens in foods.

Isothermal nucleic acid amplifications are also being incorporated in lab-on-a-chip. Liu *et al.* [95] developed a disposable, water-activated, and self-heating microfluidic cartridge for isothermal nucleic acid amplification. The device is powered by an exothermic chemical reaction (“thermal battery”) and does not require any instrument and/or power. The heat is generated by reacting magnesium with water in the presence of iron. The reactor's temperature is regulated and rendered independent of ambient temperatures with the aid of a phase change material. The detection limit for *E. coli* was down to 10 target molecules in the sample. They also reported on a microfluidic cassette with a single reaction chamber for isothermal amplification and an integrated membrane for isolation, concentration, and purification of DNA/RNA [96]. The nucleic acids captured by the membrane are used directly as templates for amplification without elution, thus simplifying the cassette's flow control. Thermal control is provided with a thin film heater external to the cassette. The amplification process was monitored in real time with a portable, compact fluorescent reader.

Jenkins *et al.* [97] designed a simple handheld instrument to enable real-time detection of the LAMP reaction in a standard PCR tube using newly assimilating probes as sequence-specific reporter molecules. The system was validated using DNA isolated from *Salmonella enterica*, demonstrating accurate temperature control with little power and little overshoot of setpoint temperatures, with rapid and accurate detection often in less than 30 min and within 20 min for reactions with high (>10^5^) genome copy numbers. Wang *et al.* [98] detected *Listeria monocytogenes* using a LAMP assay. The detection limit for artificially contaminated raw milk samples by the LAMP assay was 186 CFU/mL corresponding to 8–10 cells per reaction tube, while that of conventional PCR was 1.86 × 10^5^ CFU/mL. Data on natural raw milk samples indicated that the LAMP method was highly specific and sensitive, giving 91.67% concordance with the ISO 10560 reference method for the samples without enrichment and 100% concordance after enrichment. Hara-Kudo *et al.* [99] developed a LAMP assay for rapid (within 60 min) detection of verotoxin-producing *E. coli*. The sensitivity of the LAMP assay was found to be >0.7 CFU per test using serogroups O157, O26 and O111 of verotoxin-producing *E. coli*; this sensitivity is greater than that obtained by PCR assay. Ye *et al.* [100] reported to detect *Salmonella* in artificially contaminated eggshells using *in situ* LAMP. The key feature of *in situ* LAMP is the mild isothermal conditions (*i.e.*, low reaction temperature). Compared with traditional culture methods and conventional PCR methods, the *in situ* LAMP method needs shorter experiment cycles. The *in situ* LAMP method causes less damage than the PCR-based method, and is less sensitive to various components. Additionally, the nucleic acid extraction and cell destruction are not required in this method. Therefore, *in situ* LAMP constitutes a potentially valuable tool for rapid detection of foodborne pathogens.

The challenges of these isothermal nucleic acid amplification techniques include: (1) complications in primer design (e.g., LAMP requires 4–6 specific primers that are difficult to design for new users); (2) off-chip manipulations are often necessary; and (3) the final product is typically a complex mixture (e.g., LAMP generates stem-loop cauliflower-like DNA structures of various sizes) [101].

## 6. Cell Manipulations and Culturing in Lab-on-a-Chip

Since cell culture (and subsequent colony counting) is one of three standard methods of detecting foodborne pathogens, one can consider such demonstrations in lab-on-a-chip platform. In fact, cell manipulations, including lysis, DNA/RNA extraction, cell counting, cell sorting, and cell-cell interaction study, have extensively been attempted in lab-on-a-chip platforms, towards detecting foodborne pathogens. However, culturing cells for quantification in a lab-on-a-chip device still remains a rarity. Lab-on-a-chip study has typically been focused on fast and miniaturized experiments, and conventional cell culturing simply takes too much time (a day or two) to be demonstrated in a lab-on-a-chip platform. Thus, cell culturing in lab-on-a-chip has been limited to the studies on proliferation of certain types of cells, including cancer and stem cells [102,103], but not for detecting foodborne pathogens.

## 7. Use of Embedded Optical Fibers or Optical Waveguide in Lab-on-a-Chip

One traditional drawback of lab-on-a-chip devices had been their high detection limit (thus poor sensitivity), which arose because the small volume diminished detectable signals. Today however, much lower detection limits (at the single-molecule level) can be obtained by using optical detection. In the last decade, biosensor researchers have begun to integrate optical fibers with lab-on-a-chip microfluidic devices to enhance their sensitivity. Typically, an optical fiber is used to deliver light from its source and another set of fiber is used for detecting bio-reactions. However, we have to bear in mind that very low detection limits using optical methods are possible only in relatively clean systems. In addition, sample volumes must be sufficient to maintain such low detection limits. For instance, one requires at least 100 μL aliquot of a sample to detect 10 CFU/mL cells, since this volume contains only one viable cell.

**Figure 15.**
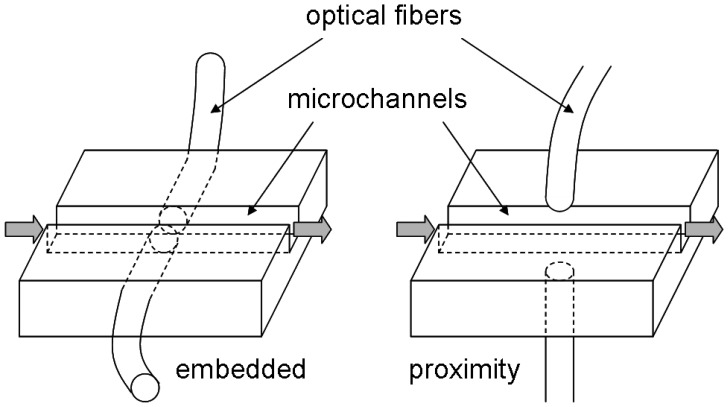
Embedded and proximity optical fibers for lab-on-a-chip. Reprinted from [47] with permission © American Society for Agricultural and Biological Engineers.

In general, the use of optical fibers in lab-on-a-chips falls into two categories of fiber orientation, as shown in Figure 15: (1) embedded fibers; and (2) proximity fibers. Embedded fibers are actually incorporated within a lab-on-a-chip device with physical contact to microchannel structures. This strategy offers the best performance with insignificant signal loss. However, it does require more complicated fabrication processes.

Proximity fibers, on the other hand, are located in close proximity to, but not touching the chip. Thus, they are easy to develop, although they may introduce additional noise in their signals. Using this setup, Han *et al.* have demonstrated the ability to detect *E. coli* at a single-cell level [53]. Proximity fiber configuration has been suggested as an economical yet highly sensitive alternative, although the entire configuration can become bulky and difficult to operate.

**Figure 16.**
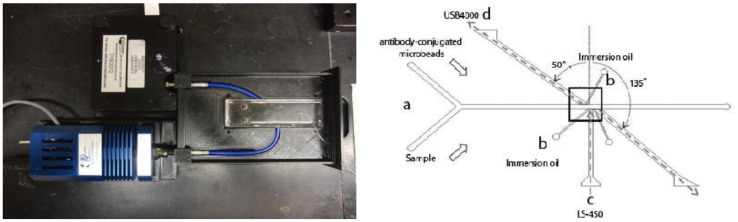
The lab-on-a-chip with optical waveguide channels to irradiate the main microfluidic channel and detect light scattering from it. Silicone oil within the optical waveguide channel acts as a core and the surrounding polydimethyl siloxane (PDMS) lab-on-a-chip acts as a cladding of an optical fiber. Reprinted from [107] with permission © SPIE.

Addition of a planar optical waveguide with a liquid core (to replace fiber insertion) overcomes these challenges, creating a simple method of both fabrication/assembly and operation. Biosensors (but not lab-on-a-chip devices) based on planar optical waveguides has provided versatile and robust transduction sensor platforms for the rapid and sensitive analysis of complex environmental and medical samples, with facile integration with sample delivery and detection systems [104]. A handful of techniques have recently been investigated for integrating optical waveguides onto lab-on-a-chip devices, including integrated microfluidic planar optical waveguide system for measuring light scattered from a single scatterer [105], and liquid core optical waveguides for DNA fragment analysis [106]. Integrated optical waveguides have not been investigated previously for use in realistic biological assays, especially general immunoassays or latex agglutination tests. However, the technology is a good fit for the application, as it allows for precise positioning of the light source and detector at a very close proximity to the detection area of the chip, which eliminates the need for larger and expensive optical positioning stages. Angus *et al.* [107] recently demonstrated the use of such integrated optical waveguides in lab-on-a-chip for detection of *Cryptosporidium* from various field water samples (Figure 16).

## 7. Conclusions

Once all the requirements addressed in this review are demonstrated for the lab-on-a-chip sensors towards foodborne pathogens, including extremely high sensitivity, rapidness and portability, it will enable us to monitor early spread of such pathogens in food and water before the outbreaks can occur (current practice is backwards—detections are made after the outbreaks). This new type of practice will save costs associated with foodborne and waterborne illness. In US, this cost is estimated to be $152 billion, according to a recent study by Pew Charitable Trusts and Georgetown University [108]. The proposed system also has a potential to be used in other applications, for example, medical diagnostics.

## Figures and Tables

**Table 1.** Summary of detection limits for various types of immunoassay lab-on-a-chips for foodborne pathogens.

**Format**	**Target & Sample Matrix**	**Detection Limit**	**Ref.**
LFA	IgG in mosquito bloodmeal *Schistosoma* in urine *Streptococcus pneumonia* in fish sperm *Treponema pallidum* in serum	10 ng/mL 0.2 ng/mL 1 ng/mL 5 ng/mL	[32] [33] [34] [35]
ELISA LOC	*E. coli* O157:H7 Staphylococcal enterotoxin B *Salmonella*	3.2 × 10^1^ CFU/mL 0.5 ng/mL 10^3^ CFU/mL	[36] [37] [38]
SPR	Antibiotics in milk *E. coli* O157:H7 in milk, juice & beef *E. coli* O157:H7 *E. coli* O157:H7 in apple juice *Salmonella typhimurium* in apple juice *Salmonella typhimurium*	1.1–2.1 ng/mL 10^2^–10^3^ CFU/mL 10^4^–10^6^ CFU/mL 10^4^ CFU/mL 10^4^ CFU/mL 10^2^ CFU/mL	[42] [43] [44] [45] [45] [46]
LIA LOC	Avian influenza in chicken feces *E. coli* in lettuce *Salmonella* in chicken tissue	10 pg/mL 10 CFU/mL 10 CFU/mL	[58] [59] [60]
IME LOC	*E. coli* in lettuce Salmonella in milk *E. coli*	10^4^–10^6^ CFU/mL single cell w/ culturing 10^2^ CFU/mL	[61] [62] [63]
CNT LOC	*E. coli* O157:H7 T7 bacteriophage Staphylococcal enterotoxin B in food Hepatitis B in human serum	10^3^–10^5^ CFU/mL 10^3^ PFU/mL 10 pg/mL 10 pg/mL	[64] [64] [65–67] [68]
